# The Correlation Between Ankle Somatosensory Acuity and Sensory Organisation in Postural Stability

**DOI:** 10.1177/00315125251343158

**Published:** 2025-05-16

**Authors:** Ashleigh Marchant, Jeremy Witchalls, Sarah B. Wallwork, Nick Ball, Gordon Waddington

**Affiliations:** 1 Research Institute for Sport and Exercise, 110446 University of Canberra, Canberra, ACT, Australia; 2 IIMPACT in Health, 1067 University of South Australia, Adelaide, SA, Australia

**Keywords:** active movement extent discrimination apparatus (AMEDA), sensory organisation test (SOT), postural stability, somatosensation, ankle somatosensory acuity, balance ergonomics motor skills & ergonomics

## Abstract

The sensory organisation test (SOT) and active movement extent discrimination assessment (AMEDA) are commonly used tools to assess postural stability and somatosensory acuity. Research on the relationship between these assessments is limited. This study aimed to explore the relationship between ankle somatosensation and postural stability in healthy adults. Participants completed one assessment of ankle somatosensory acuity (AMEDA) and one assessment of postural stability (SOT). Ankle somatosensory acuity was assessed on the non-dominant foot and measured their ability to detected small changes in joint movement within the inversion/eversion plane. The SOT involved both feet upon the testing platform and six “conditions” which distorted the sensory systems and assessed the ability to use visual, somatosensory, and vestibular feedback to maintain postural control. A Spearman’s Rank-Order Correlation was run to assess the relationship between AMEDA and SOT measures. We hypothesised that AMEDA scores would positively correlate with SOT conditions 4–6 (sway-referenced platform for all) and the somatosensory (SOM) sensory score. 54 participants (28 females, 26 males; mean age 40 ± 14 years) completed the study. Positive correlations were found between the AMEDA score and SOT conditions 5 (eyes closed, sway-reference platform) and 6 scores (sway-referenced visual surround and platform) (*p* = .041 and *p* = .006) but not with SOT condition 4 (eyes open, sway-referenced platform) or the SOM sensory score (*p* > .05). There were positive correlations between the AMEDA score, and SOT composite score and vestibular (VEST) sensory score (*p* < .001 and *p* = .007). Somatosensation and postural stability scores were related during the most challenging balance tasks, highlighting the role of somatosensory acuity in postural control. However, AMEDA score did not relate to the SOM scores in the SOT, suggesting different factors influence these measures of somatosensation. This highlights the unique contributions of the AMEDA and SOT in assessing sensory function and its impact on balance.

## Introduction

Balance is a commonly used term to describe postural control and stability, and can be described as maintaining one’s centre of gravity in a state of equilibrium within their base of support (Pollock et al., 2000). Balance is achieved by using constant adjustments of muscles and joints directed by sensory feedback (Greve et al., 2007). It relies on adequate input from the visual, vestibular, and somatosensory systems to respond with the appropriate motor commands and coordination (Greve et al., 2007; Pollock et al., 2000). Poor balance can negatively impact many aspects of human movement and increase the risk of falls (Brown et al., 2015). Good balance, or adequate postural control and stability, is therefore important for good quality of life.

The sensory organisation test (SOT) is an assessment tool frequently used to evaluate balance by measuring the contribution of different sensory mechanisms to an individuals’ postural stability. The SOT calculates maintenance of postural control and use of the visual, somatosensory, and vestibular systems by manipulating the patient’s environment through modification of visual and somatosensory feedback (Trueblood et al., 2018). By eliminating components of sensory information (e.g., eyes closed to remove visual feedback) and replacing it with misleading information (e.g., an unstable support surface), the SOT determines how well the individual can overcome conflicting sensory cues, adjust to sensory reweighting, reduce body sway, and maintain postural stability (Henry et al., 2022; Trueblood et al., 2018).

Somatosensation, one of the main sensory aspects of balance control, is comprised of proprioception (the detection of joint position and movement) and tactile sensation (the detection of pressure on the skin) (de Haan & Dijkerman, 2020). Somatosensation enables identification of the location of one’s limbs in space and in relation to the nearby environment. It is important for maintaining upright posture during locomotion and while completing activities in daily living (Kars et al., 2009). Good somatosensory acuity has been associated with enhanced athletic performance (Lin et al., 2006), while joint instability and/or musculoskeletal injury can negatively affect somatosensory acuity of the affected body region (Ingersoll et al., 2008; Witchalls et al., 2014). Joint instability has been shown to not only impair joint position sense (i.e., proprioception; a subsection of somatosensation) but has also been strongly correlated with reduced static balance (Lee et al., 2006; Waddington & Adams, 1999).

The active movement extent discrimination assessment (AMEDA) is a protocol used to evaluate somatosensory acuity. It utilises active movements by the participant and requires the that the participant detects and discriminates between small changes in the extent of a joint range of movement (Waddington & Adams, 1999). Most commonly used at the ankle, the AMEDA has also been applied to other joints around the body including the knee, spine, shoulder, and finger joints (Han, Waddington, et al., 2015). A high score on the AMEDA indicates greater somatosensory acuity while a low score is indicative of poor somatosensory acuity. Higher sporting ability is associated with higher ankle AMEDA scores while ankle injury is associated with lower ankle AMEDA scores (Steinberg et al., 2019). The AMEDA has been used to assess somatosensory acuity in a variety of populations including military personnel (Witchalls et al., 2021), athletes (Han, Waddington, et al., 2015), individuals with chronic ankle instability (Han et al., 2022), children (Yang et al., 2019), and older adults (Yang et al., 2019). While the AMEDA has been conducted alongside the SOT (Antcliff et al., 2023; Ma et al., 2020), the relationship between the ankle AMEDA and the SOT within a healthy adult population has not yet been explored.

Understanding the relationship between the AMEDA and SOT could reveal whether the tasks complement one another, predict performance in somatosensory acuity and postural stability, or open avenues for targeted interventions. Although both tasks measure somatosensory ability, they are based on different principles with distinct underlying physiological mechanisms. The AMEDA is a functional assessment of proprioception that uses a forced-choice task. It evaluates one’s ability to determine joint position across discreet changes in joint angle (Waddington & Adams, 1999). In contrast, the SOT is a comprehensive assessment of postural stability, which includes assessing the ability to adapt to varying sensory input and maintain balance (Trueblood et al., 2018). While the AMEDA is useful for focussing on a specific area of the body (e.g., ankle), the SOT provides a broader assessment of balance ability and does not isolate function to the same degree of the AMEDA. While several techniques are available to assess somatosensory acuity, the AMEDA allows participants to discriminate between small changes within a joint, in an active, self-driven manner (as opposed to passive assessment techniques) (Han et al., 2016). Unlike the SOT, the AMEDA is a unilateral task, suggesting limited interhemispheric transfer of somatosensory information. However, the AMEDA is valuable for identifying specific joint deficits or areas for improvement (Han et al., 2022; Steinberg et al., 2019; Witchalls et al., 2014). Similarly, while other methods to assess postural control exist, the SOT is regarded as the gold standard for balance performance (Wagner and Merfeld, 2003). It offers a straightforward procedure for understanding how individuals interpret misleading sensory information and adapt through sensory reweighting (Sung & Rowland, 2024).

Both the AMEDA and SOT utilise a moveable platform, and understanding performance on the AMEDA compared to performance on the SOT, could enhance our understanding of how an individual integrates sensory information. For example, high joint somatosensory acuity on the ankle AMEDA may reflect greater ability to detect changes in ankle movement and overcome misleading sensory input within the SOT. Alternatively, it could indicate a greater reliance on somatosensory signals to maintain stable posture. It is unclear whether the changes in joint angles presented by the ankle AMEDA are correlated with the platform distortions produced within the SOT. By exploring the relationship between these two tasks, we can gain a more comprehensive understanding of how ankle somatosensory acuity and postural stability interact, potentially leading to more effective assessments and interventions in clinical practice.

The aim of this study was to investigate the relationship between ankle somatosensation and postural stability in a healthy adult population. Specifically, we hypothesised that there would be a significant positive correlation between AMEDA scores and SOT test conditions 4–6 (sway-referenced platform for all) and the SOM sensory score. The SOT involves six testing conditions, presented to the participant in a sequential order, which produces a series of equilibrium and sensory scores (Henry et al., 2022; Summers et al., 2022). Conditions 4–6 involve a moveable platform which distorts somatosensory feedback and requires delicate ankle adjustments to maintain postural stability. The SOM sensory score is a direct measure of the patient’s ability to use somatosensory feedback to maintain postural control. A positive correlation between ankle AMEDA and these SOT scores would suggest (i) the tasks assess somatosensation in the same manner, (ii) greater ankle somatosensory acuity is associated with sensory reweighting, and (iii) ability (or inability) to discriminate between fine ankle adjustments within the AMEDA contributes to the ability to maintain postural control on the SOT.

## Method

### Participants

Fifty-five healthy adult participants were recruited for this study. The data used in this study is a subset from a larger project which was registered with the Open Science Framework prior to data collection (Marchant, Wallwork, Ball, Witchalls, & Waddington, 2023). Inclusion criteria were adults between 18 and 65 years of age, could understand and speak English, and considered themselves healthy and unrestricted (i.e. had the ability to move without any restrictions that impacted day to day tasks). Exclusion criteria were any condition which may affect balance (e.g., diminished sensation, inner ear loss of function), an ankle injury within the previous three months, or an inability to complete all tasks. The study was approved by the University of Canberra Human Research Ethics Committee (reference number: 202312043).

### Procedure

All participants attended the laboratory for a single 60-minute session. Written informed consent was obtained prior to testing. Participants completed a basic demographic questionnaire (including age, sex, height, weight, and preferred kicking foot) and the Cumberland Ankle Instability Tool (CAIT). The CAIT was administered to determine the presence of chronic ankle instability. Previous research has indicated individuals with ankle instability (but are otherwise healthy), have poorer ankle somatosensory acuity, as measured via the AMEDA, compared to those without (Witchalls et al., 2014). Participants then completed an assessment of ankle somatosensory acuity via the ankle AMEDA task and an assessment of balance via the SOT. Previous research has demonstrated that performance on the ankle AMEDA may be influenced by tasks that fatigue the muscles around the ankle, especially in individuals with chronic ankle instability (Steinberg et al., 2023). To reduce the confounding impacts of this, all participants completed the AMEDA protocol prior to the SOT task.

### Active Movement Extent Discrimination Assessment (AMEDA)

**Figure 1.**
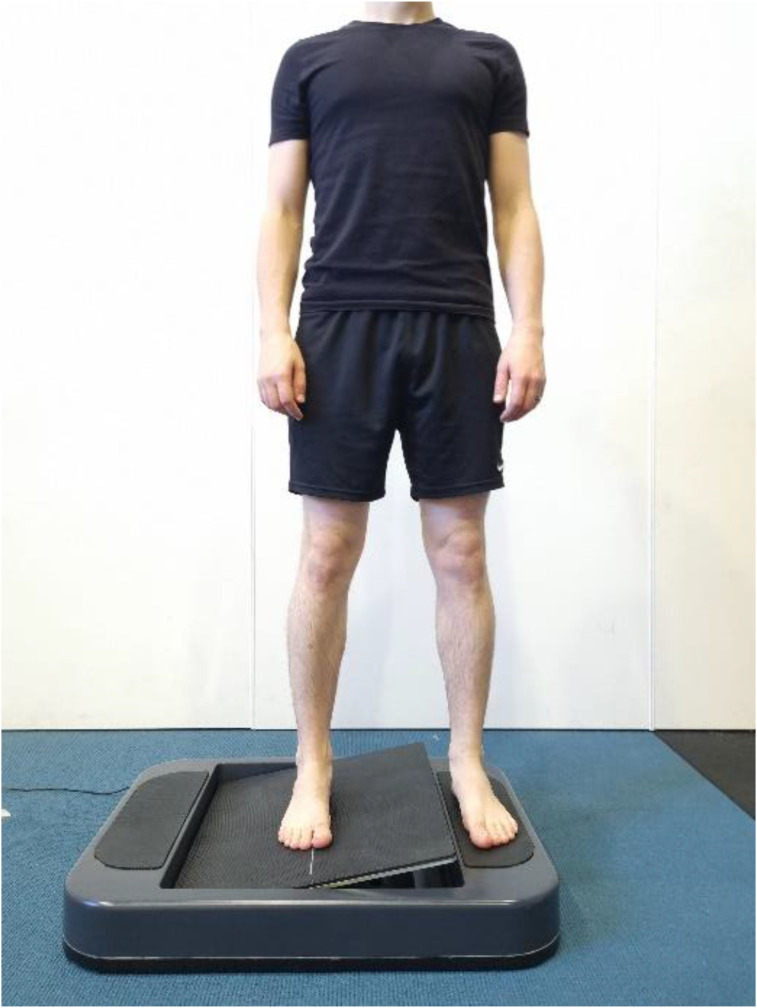
Participant completing the Active Movement Extent Discrimination Assessment (AMEDA) with their right foot on the testing platform. During the task, the participant was asked to move the platform (testing foot) by inverting their right ankle as far as the platform would allow (rigid endpoint) to one of five pre-defined depths.

The ankle AMEDA was used to assess participants ankle somatosensory acuity. Participants stood on the device, with their testing foot upon a moveable platform and their non-testing foot on a stationary platform (Figure 1). Testing was completed on their non-dominant foot (non-kicking leg) as this has been shown to have better somatosensory acuity than their dominant foot (kicking leg) (Han et al., 2013). The moveable platform rotated into five extents (depths) of ankle inversion (between 10.5 and 14.5° from the horizontal, i.e., at 1-degree increments). Participants controlled the velocity of movement and were asked to tip the platform (moving their ankle into inversion) until the platform stopped and then return to neutral (horizontal platform position). The five depths of inversion were presented to the participant prior to the formal assessment three times in sequential order to familiarise themselves with the various inversion depths. The formal assessment then commenced, and the participant was presented with the 5 inversion depths in a pseudorandom order (10 occasions at each depth, 50 in total). For each movement they indicated the position (1 to 5) they felt they had experienced. Participants were asked to maintain their gaze directly ahead with their arms by their side throughout the task. Participant responses were recorded manually by the principal investigator via an Android tablet and uploaded to a Microsoft Excel spreadsheet (Microsoft ® Corporation. 2023. Version 2301. Retrieved from https://office.microsoft.com/excel). A matrix of responses was generated to form an area under the curve (AUC) score of a Receiver Operating Characteristic (ROC) curve, with potential scores between 0.5 (equivalent to chance) and 1.0 (a perfect score).

### Sensory Organisation Test (SOT)

**Figure 2.**
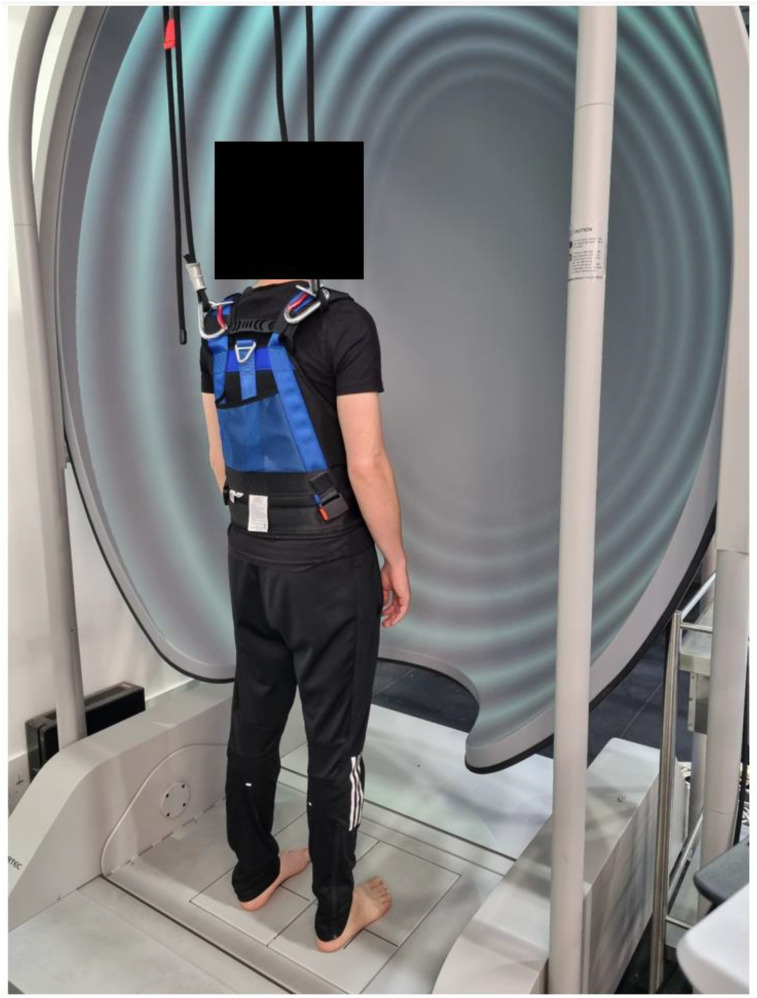
Participant standing within the Bertec® system ready to complete the sensory organisation test (SOT). During the task participants were required to maintain quiet stance as the force plate recorded their body sway.

The SOT was completed using the Bertec® Advantage Balance system® (Bertec Incorporated. 2014. https://www.bertec.com/. Columbus, OH, USA.). Previous research on the SOT has demonstrated poor to moderate within session reliability (composite score: ICC = 0.72–0.84; test condition equilibrium scores and sensory ratio scores: ICC<0.5–0.85) (Summers et al., 2022). Participants stood barefoot within a dome-shaped immersive screen (180-degree horizontal field of view and 90-degree vertical field of view) on dual force plates with their malleoli positioned in line with the horizontal lines on the plates (also the platform’s rotational axis) (Figure 2). A safety harness was worn throughout testing which only provided support in the event of a complete loss of balance. Participants were asked to focus their gaze straight ahead (in the eye-open conditions) with arms relaxed by their side.

Participants were exposed to six conditions which progressively challenged their ability to maintain quiet stance (postural stability) (see Table 1 for an outline of the 6 conditions). Each condition (i.e., 1–6) was presented three times consecutively (duration: 20 seconds each) before progressing to the next condition. Test conditions were presented in order from 1 thorough 6 to provide a gradual increase of challenge, controlled progression and ability to establish a baseline to compare the more challenging conditions with (Wrisley et al., 2007). As the test progressed, various sensory feedback was either removed or altered to increase the challenge of maintaining postural stability. For example, conditions 2 and 5 involved removal of visual input (eyes closed). Condition 3 involved conflicting sensory input of the visual system, where a sway-referenced system cued via the force applied to the platform through the feet, caused moving lines to be projected on the screen. In conditions 4 and 5, the same sway-reference system was used to cause the foot plate to swing, thereby creating an unstable standing surface distorting somatosensory input. In condition 6, both the visual and somatosensory systems were distorted as the sway-reference system induced both shifting lines upon the screen, and a moveable platform beneath the feet, thereby shifting the reliance to the vestibular system to maintain postural control. Throughout all conditions, the foot plate recorded anterior-posterior body sway which then provided a series of computer-generated scores including one composite score, 18 equilibrium scores, and four sensory scores.

**Table 1.** The six conditions of the SOT. Each condition was Presented to the participant Three Times for 20 seconds each Before Progressing to the Next one.

Condition	Vision	Surface	Visual surround	Intended Sensory Input
1	Eyes open	Stable	Stable	Visual, vestibular, somatosensory
2	Eyes closed	Stable	-	Vestibular, somatosensory. Visual removed
3	Eyes open	Stable	Sway-referenced	Vestibular, somatosensory. Visual distorted
4	Eyes open	Sway-referenced	Stable	Visual, vestibular. Somatosensory distorted
5	Eyes closed	Sway-referenced	-	Vestibular. Visual removed. Somatosensory distorted
6	Eyes open	Sway-referenced	Sway-referenced	Vestibular. Visual, and somatosensory distorted

An ‘equilibrium score’, which represents the participants ability to remain stable, was generated for each test of each condition (i.e., 18 equilibrium scores in total). This was calculated as a percentage of the participants’ centre of pressure (through their feet) within the limits of postural stability (Wrisley et al., 2007). A score of 100% is indictive of perfect postural control and a score of 0% indicates complete loss of balance. The ‘composite score’ is a weighted average of all equilibrium scores accounting for the increasing difficulty of conditions (Henry et al., 2022; Wrisley et al., 2007).

The ‘sensory scores’ are generated as ratios of the six conditions used to assess how the participant relied on the various sensory (visual, somatosensory, vestibular) cues for their balance (Henry et al., 2022). The visual (VIS) sensory score is a ratio of condition 4/condition 1, which determines how much the participant relies on visual cues. The somatosensory (SOM) sensory score is a ratio of condition 2/condition 1, which determines how much the participant relies on somatosensory input. The vestibular (VEST) sensory score is a ratio of condition 5/condition 1, which determines how much the participant relies on vestibular input. The visual preference (PREF) sensory score is a ratio of the sum of conditions 3 and 6, to the sum of conditions 2 and 5. Henry et al. (2022) describe this as how well the individual has ignored inaccurate visual cues, as it assessed how the participant relies on visual information even if the visual cues were misleading. For example, if a participant had a low PREF score, they were likely predominantly dependant on visual information for postural stability despite the visual feedback being unreliable (Perucca et al., 2021).

### Data Analysis

SPSS statistics (IBM Corp. Released 2023. IBM SPSS Statistics for Windows, Version 29.0. Armonk, NY: IBM Corp) was used to analyse all results. The data were tested for outliers by inspecting boxplots, where any data points located more than 1.5 times the interquartile range (IQR) from the edge of the box (i.e., beyond the whiskers) were considered outliers, as flagged by SPSS Statistics. Prior to undertaking our main analysis, we conducted an independent samples t-test comparing AMEDA scores between people with and without ankle instability, due to the known influence of ankle instability on somatosensory acuity (Waddington & Adams, 1999; Witchalls et al., 2014). For completeness, an independent sample t-test was also conducted on SOT composite scores between people with and without ankle instability. If a difference in performance was found between groups, participants with ankle instability were excluded from subsequent analyses. We also conducted a one-way analysis of variance (ANOVA) to determine whether body mass index (BMI) influenced AMEDA and SOT composite scores, as high BMI can be associated with poor proprioceptive control at the ankle (Cho et al., 2023). Participants were classified into three groups; group 1: BMI between 18.5–24.9; group 2: BMI between 25–29.9; group 3: BMI of 30 or above.

Ankle AMEDA (AUC) scores and SOT scores (composite, the best of equilibrium score for conditions 1–6, and sensory scores VIS, SOM, VEST and PREF) were tested for normality via a Shapiro-Wilk’s test. Not all variables of the SOT were normally distributed (*p* < .05). Spearman’s Rank-Order Correlations were therefore used to assess the relationship between AMEDA scores (AUC) and SOT scores. Visual inspection of a scatterplot was used to determine whether the relationship between variables was monotonic prior to analyses. Strength of the relationship was guided by Akoglu (2018), where a correlation coefficient of <0.2 was considered poor, 0.3–0.5 was considered fair, 0.6–0.7 was considered moderate, and >0.8 was considered very strong. An alpha value of 0.05 was used to determine statistically significant results.

## Results

Fifty-five participants were recruited for this study however one was unable to complete conditions 5 and 6 of the SOT and was therefore removed from analyses. For the remaining 54 participants (28 females, 26 males), the mean and standard deviation of age, height, and weight were 40 years ± 14, 173 cm ± 9, and 76 kg ± 13. Forty-eight considered their right foot to be their preferred kicking foot. There were two outliers among the AMEDA data. These were kept in analysis as both were considered high scores but not unusual (AMEDA AUC score 0.80 and 0.82). There were no outliers in the SOT composite, condition 1, condition 2, condition 5 and VIS sensory score. There were outliers in SOT condition 3 (*n* = 1), condition 4 (*n* = 4), condition 6 (*n* = 1), SOM sensory score (*n* = 3), VEST sensory score (*n* = 1), and PREF sensory score (*n* = 2). This was anticipated due the poor to moderate within session reliability of the SOT (Summers et al., 2022).

Thirteen participants were classified as having chronic ankle instability, however an independent t-test indicated that there was no difference in AMEDA scores, or SOT composite scores, between participants with and without ankle instability: *t*(52) = −1.53, *p* = .133; *t*(52) = 0.252, *p* = .802. Therefore, subsequent analyses included the entire group (*n* = 54). There was no significant difference between BMI groups (group 1: *n* = 27, group 2: *n* = 19, group 3: *n* = 8) for AMEDA scores: F(2, 53) = 0.324, *p* = .725, and SOT composite scores: F(2,53) = 0.215, *p* = .807. Participants considered themselves to be healthy and unrestricted, which was part of the inclusion criteria, and this may explain why chronic ankle instability and BMI, factors often considered limiting, did not influence this group. Further, participants had completed the AMEDA prior to the SOT to reduce the implications of fatigue.

**Figure 3.**
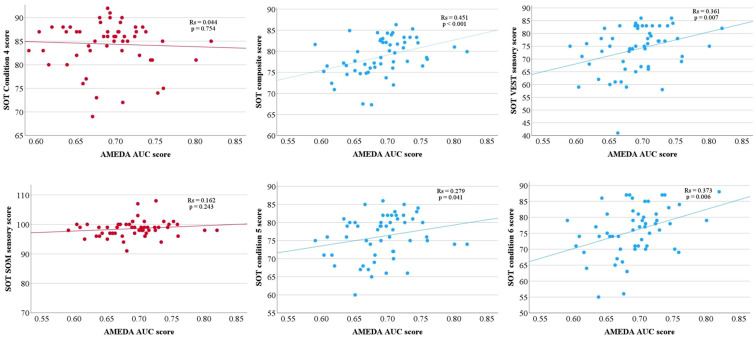
Contrary to our hypothesis, red scatter plots (left) show no significant correlations between the AMEDA AUC score and SOT scores for condition 4 and SOM sensory score (*p* > .05 for both). Blue scatter plots (middle and right) show fair positive correlations (Akoglu, 2018) between the AMEDA score and the SOT scores for composite score, conditions 5 (eyes closed, sway-reference platform) and 6 (sway-referenced visual surround and platform) scores, and VEST sensory score (*p* < .05 for all).

Of the 54 participants, the relationships between AMEDA scores and SOT scores were considered monotonic. There were significant fair positive correlations between the AMEDA score and SOT scores for: composite score (*p* < .001), conditions 5 (eyes closed, sway-reference platform; *p* = .041) and condition 6 scores (sway-referenced visual surround and platform; *p* = .006), and VEST sensory score (*p* = .007) (Figure 3). There were no significant correlations between the AMEDA score and SOT scores for: conditions 1-4 scores, and sensory scores of SOM, VIS, and PREF (*p* > .05 for all). The mean and standard deviations of each variable with the correlation coefficient and *p* values from the Spearman’s Rank-Order Correlation are shown in Table 2.

**Table 2.** A Total of 54 participants Were Included in Analysis.

	Sensory assessment	Score M ± SD	Spearman’s rho R_s_	*p* value
AMEDA	Somatosensory acuity	0.69 ± 0.05	-	-
Composite	Weighted average of conditions 1–6	78.8 ± 4.4	0.451**	<0.001**
Condition 1	All sensory systems available	94.3 ± 1.6	−0.066	0.635
Condition 2	Vestibular, somatosensory available. Visual removed	92.9 ± 2.3	0.148	0.286
Condition 3	Vestibular, somatosensory available. Visual distorted	92.7 ± 2.9	0.080	0.565
Condition 4	Visual, vestibular available. Somatosensory distorted	84.4 ± 5.2	0.044	0.754
Condition 5	Visual removed. Vestibular, somatosensory distorted	76.2 ± 6.0	0.279**	0.041**
Condition 6	All sensory systems distorted	75.8 ± 7.7	0.373**	0.006**
SOM	Reliance on somatosensory feedback	98.6 ± 2.8	0.162	0.243
VIS	Reliance on visual feedback	86.2 ± 6.4	0.108	0.435
VEST	Reliance on vestibular feedback	73.8 ± 9.1	0.361**	0.007**
PREF	Reliance on visual cues even with distorted visual feedback	99.5 ± 6.7	0.163	0.238

Test Mean and Standard Deviations (M ± SD) of the AMEDA Score, SOT Composite Score, six SOT condition Scores, and four Sensory SOT Scores are Presented, With Spearman’s rho Correlation Coefficient (R_s_) and *p* Value. AMEDA = AUC Score (Possible 0.5–1.0; a higher Score Represents Greater somatosensory Acuity); Composite = Score is a Weighted Average Across all conditions (Possible 0–100; a higher Score Represents Greater Postural Stability); Conditions 1–6 = an Equilibrium Score Represented as a Percentage (Possible 0-100%; a higher Score Represents Greater Postural Stability); VIS = visual Sensory Score, SOM = somatosensation Sensory Score, VEST = Vestibular Sensory Score, PREF = Preference Sensory Score (Ratios of the Various conditions; a higher Score Represent Greater Reliance of the Associated System). ** Denotes a Statistically Significant result. All Statistically Significant Spearman’s rho Correlation Coefficients Were Between 0.279 and 0.451 Suggesting a Fair Relationship to the AMEDA Score (Akoglu, 2018).

## Discussion

We investigated the relationship between ankle somatosensory acuity and postural stability in healthy adults. Our hypothesis was partially supported in that ankle AMEDA scores were positively correlated with conditions 5 (eyes closed, sway-reference platform) and 6 (sway-referenced visual surround and platform) of the SOT. However, the hypothesis was not supported with condition 4 (eyes open, sway-referenced platform) and the SOM sensory score where there was no positive correlation found. The AMEDA score was also positively correlated with the VEST sensory score and the composite score. It is important to note that these findings demonstrate only a fair relationship between the assessments. Further, the correlation between AMEDA and SOT composite score, is likely to be the only relationship not subject to Type 1 error. Including a Bonferroni adjustment could have strengthened the interpretation of results but equally may risk overlooking some meaningful findings (VanderWeele & Mathur, 2019). It is also reasonable to expect that the similar patterns of correlation to condition 5, 6 and VEST sensory score are less likely to be a result of Type 1 error, given the structure of the SOT scoring system, where all SOT scores are derived from a single test and the composite score is weighted toward the more challenging conditions. Overall, the results suggest that ankle somatosensory acuity is likely to be weakly associated with postural stability, and the two tasks (AMEDA and SOT) appear to differ in the techniques they use to assess somatosensation.

Participants who exhibited higher scores of somatosensory acuity, as measured using the AMEDA, also performed better at the more challenging SOT conditions (conditions 5: eyes closed, sway-reference platform, and 6: sway-referenced visual surround and platform); suggesting that higher acuity in the inversion/eversion movements is associated with greater postural stability in more challenging contexts. Although we are unable to determine whether participants of the current study relied on somatosensation more than visual and vestibular feedback in conditions 5 and 6, it is well-established that the somatosensory system plays an important role in postural control when the sensory (visual, somatosensory, vestibular) systems are challenged, highlighting the process of sensory reweighting. For instance, healthy older adults (>65years) rely more on somatosensory cues than visual or vestibular cues, compared to younger adults (<65years) for balance control during an ankle perturbation task, despite a general deterioration of all sensory (visual, somatosensory, and vestibular) systems with age (Pasma et al., 2015). Adults aged 50 and 75 years of age who exhibit heightened falls risk are more effective at somatosensory reweighting to prevent having a fall compared to those those with low falls risk (Sung & Rowland, 2024). Similarly, astronauts exposed to microgravity, show a greater reliance on somatosensory cues over vestibular input during a modified SOT, even under manipulated conditions affecting both (somatosensory and vestibular) systems (Ozdemir et al., 2018). The body’s reliance on somatosensory information varies depending on the level of available sensory input (Peterka, 2002). In children whose fine and gross motor skills are still developing, somatosensory function plays a vital role in motor skills and ability to meet physical milestones, yet they typically display poorer AMEDA scores than healthy adults whose postural control is likely to be stable (Taylor et al., 2016; Yang et al., 2019). Older adults (>75 years) also tend to score lower on the AMEDA. This is associated with increased falls risk and poorer SOT performance from age 60 onward (Antcliff et al., 2023; Whitney et al., 2006; Yang et al., 2019). Interventions aimed at enhacing ankle somatosensory acuity, by increasing somatosensory stimulation such as through external tactile sensation (Broatch et al., 2021; Marchant, Wallwork, Ball, Witchalls, & Waddington, 2023), could potentially improve postural stability and balance in populations whose somatosensory acuity is comprimised. However, as the current study was inclusive of healthy adults, further investigation is warrented as it remains unclear whether a similar trend between the AMEDA and SOT would still be present among other populations.

The most notable correlation observed in the current study was between ankle AMEDA scores and the SOT composite score, with a Spearman’s rho value of 0.451. It is important to note that this does not imply any causative relationship between the AMEDA and SOT measures (Akoglu, 2018), however, it does appear that a greater ability to discriminate between joint angles may be beneficial for maintainence of postural control when sensory systems are unreliable. The composite score is a weighted average of the equilibrium scores (conditions 1–6) with more emphasis on the more challenging aspects of the task. Often used as a primary outcome measure, the composite score is a technique used to quantitatively measure postural stability and assess one’s ability to integrate visual, somatosensory, and vestibular feedback (Grove et al., 2021; Henry et al., 2022; Trueblood et al., 2018). Our results show that although not a strong relationship, higher somatosensory acuity at the ankle is correlated to greater postural stability and ability to challenging balance conditions. Interestingly, this contradicts a previous study that found ankle proprioception did not play a significant role in stability during the gait cycle. Instead, greater hip proprioception was associated with better postural stability (Qu et al., 2022). The ankle was selected for the current study due its known role in balance, however the ankle AMEDA directly evaluates ankle somatosensory acuity through tactile and proprioceptive feedback, allowing participants to identify the degree of ankle inversion (Han, Anson, et al., 2015). This directs their focus specifically to the ankle. In contrast, the SOT evaluates postural stability within the broader context of balance and sensory organisation, which includes somatosensation but in a different manner. By manipulating the visual and somatosensory inputs, the participants’ attention during the SOT is not only on ankle placement but also to maintain balance to prevent a fall, achieved through sensory reweighting (Sung & Rowland, 2024). During external perturbations, such as those occurring in the SOT, the contribution of the visual, somatosensory, and vestibular system to maintain balance, will vary (Peterka, 2002; Sung & Rowland, 2024). This highlights the distinct roles and contributions of each task (AMEDA and SOT) in assessing sensory function.

We did not expect AMEDA scores to correlate with SOT conditions 1–3 (stable platform for all), and this was supported by the results of the study. Somatosensory feedback is intended to be available and accurate during conditions 1–3 as the SOT platform is stationary. As somatosensation includes an intricate blend of proprioception and tactile sensation, there are multiple assessment tasks routinely used to interrogate the different components of somatosensory ability (de Haan & Dijkerman, 2020; Han et al., 2016). For this reason, there is no one standard way to assess somatosensation and various assessment approaches may not necessarily correlate (Horváth et al., 2023). For example, assessment of kinaesthesia (awareness of movement in a joint) and assessment of joint position sense (awareness of joint position in space) at the knee are not correlated (Grob et al., 2002). Similarly, ankle joint position reproduction and ankle joint position discrimination are also not correlated (Yang et al., 2020). However, Yang et al. (2022) demonstrated plantar tactile sensitivity to be strongly correlated to somatosensory acuity, and Lee et al. (2006) showed a strong correlation between ankle repositioning and static balance. The different assessment methods used in these studies may not measure the same construct of somatosensation. In the current study, conditions 1–3 of the SOT involved a stationary platform suggesting predominant feedback was from pressure under the foot rather than active proprioception. The ankle AMEDA, on the other hand, utilises the participants self-initiated active movement to enable assessment of somatosensory acuity as the ankle joint is moved through a range of ankle inversion/eversion movement extents. Therefore, it seems unlikely that these tasks (SOT and AMEDA) are assessing the same elements of somatosensation.

The absence of correlation between the ankle AMEDA scores and the SOT condition 4, which involves a sway-referenced platform to distort somatosensory feedback (eyes open), is unexpected. One potential explanation could be that the ankle AMEDA assesses ankle somatosensory acuity in the inversion and eversion movement plane, while the SOT platform moves within an ankle dorsiflexion and plantar flexion movement plane, so acuity might not be translated across the two anatomically differing movements. However, there was a correlation of the ankle AMEDA scores with conditions 5 (eyes closed, sway-reference platform) and 6 (sway-referenced visual surround and platform) where the platform was also moving. Another potential explanation could be the use of sensory reweighing in the SOT, particularly the significant role of the visual system in condition 4 (eyes open, sway-referenced platform). In a study assessing postural control using the SOT, it was found that the visual system was the preferred sensory system used by young healthy adults to maintain balance (Gaerlan et al., 2012). As vision was available (eyes open) in condition 4 of the SOT it was likely the primary driver of balance control, with less contribution from the somatosensory system.

The lack of significant correlation between the SOM sensory score of the SOT with the ankle AMEDA was also unexpected. The SOM sensory score is suggested to indicate the participants’ reliance on somatosensory feedback to maintain postural balance (Henry et al., 2022). It makes intuitive sense that a high SOM sensory score should correspond with a high ankle AMEDA score. However, a closer look at how the sensory scores are calculated reveals why they may not. The SOM sensory score is calculated by dividing condition 2 (eyes closed) by condition 1 (eyes open), both of which involve a stationary SOT platform. Hain (2021), argue it is more an assessment of how the participant manages to maintain balance when vision is taken away. As mentioned above, it might also be that the predominant sensory feedback is from pressure under the foot rather than active proprioception. Because the ankle AMEDA requires a judgment of an active ankle movement range of motion, this may explain why this (SOM sensory) score did not correlate with the ankle AMEDA scores. Therefore, both tasks (SOT and AMEDA) assess different aspects of the somatosensory system.

An unexpected finding of the current results is that the VEST sensory score of the SOT exhibited a significant positive correlation to ankle AMEDA scores. The VEST sensory score, calculated by dividing condition 5 (eyes closed, sway-reference platform) by condition 1 (eyes open, stable platform), is intended to evaluate the participants balance management with no visual input (eyes closed) and confusing somatosensory input from their feet and ankles (moveable platform) thereby relying more on vestibular input (Hain, 2021). However, in the current study, participants who demonstrated good somatosensory acuity on the AMEDA, were likely to score well in conditions 5 of the SOT and therefore score well in the VEST sensory score of the SOT. Hain (2021) have noted that the sensory scores (VIS, SOM, VEST, PREF) of the SOT are derived from “noisy” equilibrium scores which may confound outcomes from this task. While the intention is to assess vestibular feedback utilisation, the VEST sensory score may also reflect the ability to adapt to confusing somatosensory feedback.

In the current study, only analysing the composite score would not necessarily demonstrate the intricacies of any relationship between the ankle AMEDA and SOT scores. The value in breaking down the composite score and analysing the six conditions separately, allowed us to differentiate how participants reacted to the increasing challenges of the SOT. Participants with higher ankle AMEDA scores, also demonstrated better performance in conditions 5 (eyes closed, sway-reference platform) and 6 (sway-referenced visual surround and platform) of the SOT. This may highlight the critical role of ankle somatosensory acuity in influencing postural control during sensory reweighting, with potential clinical implications for physical therapy and rehabilitation. However, as no Bonferroni adjustment was applied to the analysis, these results may be subject to Type I error. Additionally, the presence of outliers suggest variability within the data, which was anticipated, given that previous research has shown poor within session reliability of the SOT (Summer et al., 2022). This should be considered when interpreting this study.

### Clinical Implications and Future Directions

This study highlights the importance of somatosensory acuity in postural stability and demonstrates how different tools (i.e., SOT and AMEDA) assess somatosensation differently depending on the task context. As somatosensory acuity has the strongest relationship to postural control when balance is most challenged, future research could combine these tests with more balancing tasks such as single leg stance with eyes close or introduce head tilts during the SOT (Akgun et al., 2023; Clark & Iltis, 2008). Further, future research could assess whether interventions to increase somatosensory acuity can also impact balance in a broader context and improve postural control. Such improvement might benefit populations with visual or vestibular dysfunction, medical conditions affecting postural stability (e.g., Parkinson’s disease), the elderly, and astronauts. Additionally, the study shows how the AMEDA and SOT offer distinct assessments of sensory performance, and each provide unique insight into human performance.

### Limitations

This study has limitations. All participants completed the AMEDA before the SOT, with the intention of minimising SOT fatigue from impacting the AMEDA scores. However, this raises the possibility of an order effect. This study formed a subset from a larger project, and we cannot be certain that this influenced results, particularly fatigue or learning effects from repeat testing throughout the session. We did not consider the effect of only one SOT and future research should include at least two baseline tests to improve the within session reliability of the SOT (Dickin & Clark, 2007). Results were not adjusted to account for Type 1 error so should be interpreted accordingly. A low *p* value of 0.005 would have accounted for this and certainly would strengthen the results however could also lead to missing potential findings. We completed ankle AMEDA testing solely on the non-dominant ankle. Given the SOT assesses somatosensation in both ankles and feet simultaneously, future studies might benefit from assessing both lower limbs on the AMEDA.

## Conclusions

This study investigated the relationship between ankle somatosensory acuity and measures of postural stability in a healthy adult population. We found a “fair” positive correlation between AMEDA scores to conditions 5 (eyes closed, sway-reference platform) and 6 (sway-referenced visual surround and platform) of the SOT when the visual and somatosensory system information was most distorted. This suggests that somatosensory compensation may be crucial in situations where sensory systems and function are challenged. There was also a “fair” positive correlation between AMEDA scores, and the SOT composite score which implies the ability to detect small changes in ankle joint movement is beneficial for more stable postural control. AMEDA scores were also positively correlated to the SOT VEST sensory score which indicate that the evaluation of somatosensation differs between the two tasks, emphasising the complex system of sensory feedback that influences postural stability. Since these two tasks reveal differences in participant strategies for gathering somatosensory information and achieving postural control, both tasks have unique value to data collection in studies of postural control.

## Data Availability

Research data can be made available by the authors, without undue reservation. *
